# Towards Interoperability in Genome Databases: The MAtDB (MIPS *Arabidopsis Thaliana* Database) Experience

**DOI:** 10.1002/cfg.278

**Published:** 2003-04

**Authors:** Heiko Schoof

**Affiliations:** Technische Universität München Lehrstuhl genomorientierte Bioinformatik Wissenschaftszentrum Weihenstephan Freising 85350 Germany

## Abstract

Increasing numbers of whole-genome sequences are available, but to interpret them fully requires more than listing all genes. Genome databases are faced with the challenges of integrating heterogenous data and enabling data mining. In comparison to a data warehousing approach, where integration is achieved through replication of all relevant data in a unified schema, distributed approaches provide greater flexibility and maintainability. These are important in a field where new data is generated rapidly and our understanding of the data changes. Interoperability between distributed data sources allows data maintenance to be separated from integration and analysis. Simple ways to access the data can facilitate the development of new data mining tools and the transition from model genome analysis to comparative genomics. With the MIPS *Arabidopsis thaliana*  genome database (MAtDB, http://mips.gsf.de/proj/thal/db) our aim is to go beyond a data repository towards creating an integrated knowledge resource. To this end, the *Arabidopsis* genome has been a backbone against which to structure and integrate heterogenous data. The challenges to be met are continuous updating of data, the design of flexible data models that can evolve with new data, the integration of heterogenous data, e.g. through the use of ontologies, comprehensive views and visualization of complex information, simple interfaces for application access locally or via the Internet, and knowledge transfer across species.


**Comparative and Functional Genomics**
*Comp Funct Genom* 2003; **4**: 255–258.
Published online 1 April 2003 in Wiley InterScience (www.interscience.wiley.com). **DOI:** 10.1002/cfg.278

**Conference Review**

### Towards interoperability in genome databases: the MAtDB (MIPS *Arabidopsis thaliana* database) experience

Heiko Schoof*
*Technische Universität München, Lehrstuhl genomorientierte Bioinformatik, Freising, Germany*

*Correspondence to: Heiko Schoof, Technische Universität München, Lehrstuhl genomorientierte Bioinformatik, Wissenschaftszentrum, Weihenstephan, 85350 Freising, Germany. E-mail: h.schoof@weihenstephan.de*

Received: 29 January 2003
Revised: 5 February 2003
Accepted: 6 February 2003

### Abstract

**Increasing numbers of whole-genome sequences are available, but to interpret them fully requires more than listing all genes. Genome databases are faced with the challenges of integrating heterogenous data and enabling data mining. In comparison to a data warehousing approach, where integration is achieved through replication of all relevant data in a unified schema, distributed approaches provide greater flexibility and maintainability. These are important in a field where new data is generated rapidly and our understanding of the data changes. Interoperability between distributed data sources allows data maintenance to be separated from integration and analysis. Simple ways to access the data can facilitate the development of new data mining tools and the transition from model genome analysis to comparative genomics. With the MIPS *Arabidopsis thaliana* genome database (MAtDB, http://mips.gsf.de/proj/thal/db) our aim is to go beyond a data repository towards creating an integrated knowledge resource. To this end, the *Arabidopsis* genome has been a backbone against which to structure and integrate heterogenous data. The challenges to be met are continuous updating of data, the design of flexible data models that can evolve with new data, the integration of heterogenous data, e.g. through the use of ontologies, comprehensive views and visualization of complex information, simple interfaces for application access locally or via the Internet, and knowledge transfer across species.** **Copyright © 2003 John Wiley & Sons, Ltd.**

**Keywords: bioinformatics; genome database; data integration; data mining; database interoperability; BioMOBY**

### Introduction

Complete genome sequences and protein-coding gene sets are becoming available for a growing number of organisms. While these are proving highly informative and invaluable for studying those and related organisms, at the same time they make it clear how far we still have to go before reaching an in-depth understanding of how a genome determines the lifestyle of an organism. As we gain an overview of one level of information — the genes as the basic blocks of genetic information — the complexity of the next levels becomes clearer. This fosters a view where a genome is a listing of parts, and in order to understand how the whole mechanism works it is necessary to know which parts interact and how these interactions are regulated.

Knowledge of the parts list, i.e. of all genes of an organism, needs to be complete in order to answer questions like, 'Does this organism have a particular metabolic pathway?'. This requires predicting enzymatic functions for all genes and mapping them onto metabolic pathways. Another example is as follows: to determine the set of downstream genes that are regulated by a specific transcription factor, all genes that contain its binding site in their promoter need to be listed; additionally, other factors that might interact would be of interest, e.g. proteins that bind to the transcription factor.

Copyright © 2003 John Wiley & Sons, Ltd.

To analyse the function of this regulatory system, knockout phenotypes of the transcription factor and its downstream genes would be helpful.

These questions bring together different data types: conserved genes are analysed in the context of metabolic networks, sequence patterns are correlated with phenotypes. While the human mind is expert at bringing together heterogenous data from unrelated sources, when assessing these questions at a genomic scale, human integration becomes effectively impossible. Instead, bioinformatic tools are required that can perform the comprehensive, correlative, comparative analyses needed to bring together the knowledge hidden in the disparate data. This process of finding relations and information hidden in the data is known as 'data mining'.

Currently, the available data for different datatypes is generally stored separately in individual databases. Data models and formats are heterogenous, access is mostly via html-based web display requiring human browsing, and even collecting the data in order to integrate it by human inspection is tedious. In order to automate data integration, not only are integration tools necessary, but also the underlying data sources need to meet several requirements.

In this review, which is based on a presentation given at the PAGXI 'Bioinformatic interfaces, ontologies and interoperability' workshop, a wish-list for genome data sources will be discussed, along with interoperability solutions aimed at moving from mere gene dictionaries to integrated genome knowledge resources.

### Keeping data current

Sequence data has changed biology because it can (at least in principle) be determined exactly. A finished genome sequence of high quality contains about 1 misread nucleotide every 10 000 bp. Once a genome sequence has been finished, only very minor changes need be anticipated. However, interpreting the sequence and adding information, a process or data referred to as annotation, is based largely on predictive methods with a wide margin of error, e.g. in the *Arabidopsis thaliana* genome, at the time of publication, only about 10% of the 26 000 predicted genes could be verified by experimental evidence [6]. Scientists working with the genome data have had to cope with the fact that gene models changed as prediction methods were improved and new data, such as full-length cDNA sequence, became available [2]. Gene function prediction is primarily based on sequence similarity or domain detection. As new, characterized proteins are added to the databases, this type of annotation needs to be updated.

Manual curation of model organism databases would require massive manpower to keep up with the pace with which new data is generated. At the least, the human curators need every support that can be provided by automating this process. This leads to annotation systems such as Ensembl [3], PEDANT [1] or riceGAAS [4], where as much manual annotation as possible is preserved from one version to the next while recalculating all predictions. Through large-scale cDNA sequencing projects, the major part of *Arabidopsis* gene models can now be based on alignments between genomic DNA and full-length cDNA sequence. By automatically downloading any newly available cDNA sequence, aligning it to the genome and adjusting gene models where necessary, gene models can be continually improved.

### Evolving data models

Designing data models requires intricate knowledge of the dependencies and relations between data objects and their attributes. As knowledge of molecular genetics grows, data models to store interactions and information flow need to be adapted and new types of biological entities need to be added, or existing types modified. An interesting approach to gaining new insights into genetic information flow is to try different interaction models to assess which best fits the available data.

In order to gain the flexibility necessary to allow model evolution, one approach is to design database schemas that allow independent storage of the primary data and the semantics describing the entities, attributes and relations that are being modelled. Separation of data and data models avoids rigid structures cemented at the time of design.

### Data integration

Integrating heterogenous data requires some common point of reference between the datasets. For

Copyright © 2003 John Wiley & Sons, Ltd. 

annotation attached to sequence, sequence identity can form the basis for interrelating data, but this is not necessarily unique, and is often inconvenient. Stable, unique, globally accepted identifiers, such as the accessions of the sequence databases of the International Nucleotide Sequence Databases, are more convenient to ensure that the same thing is being referenced. Additionally, they enable versioning, so that related information need not be lost in an update. However, the version becomes critical when subsequences are being annotated, as these are generally referenced by coordinates in the sequence and these may change in an update.

For data integration purposes, the stable, unique identifiers established by the *Arabidopsis* Genome Initiative (AGI), and known as AGI gene or locus codes, have been very useful. The genome databases at TAIR (The *Arabidopsis* Information Resource, **http://www.arabidopsis.org**), TIGR (The Institute for Genomic Research, **http://www.tigr.org)** and MIPS (Munich Information Center for Protein Sequences, **http://mips.gsf.de**) have used these to cross-reference their gene reports and, based on these identifiers, more detailed analysis of specific topics like individual protein families, expression analysis or knockout experiments could be integrated with the genome databases.

Based on this principle, MAtDB (MIPS *Arabidopsis thaliana* database) [5] integrates external data by utilizing the versatility of XML. An integration tool was implemented that transforms any tabular data where one column is the AGI locus code into a generic XML format that allows the data to be displayed with the relevant MAtDB entry (**http://mips.gsf.de/cgi-bin/proj /thal/ framesetter?about&externalanno.html**).

However, not all data is gene-centric and sometimes more elaborate mappings are required. A standard for describing biological entities beyond sequences or genes and their names or identifiers would be an important step. An interesting proposition has been put forward by the BioMOBY project (**http://biomoby.org**) [7]. Here, an ontology of classes of biological objects defines what entity is being referenced. Each data object within MOBY is referenced by a triplet consisting of the class, a namespace and an identifier. The namespace defines the database or nomenclature, while the identifier is a unique, primary key for this data object.

### Comprehensive views and visualizations

Genome databases generally offer two basic views, a report that includes all information on a database entry and a graphical browser that depicts features along a sequence. But the information on a single gene is rapidly becoming too complex, and graphical displays should incorporate more dimensions than linear sequence. Intuitive displays of interactions, multiple sequence similarities, or cross-genome links need to be developed. While many ideas exist, such as circular graphs for multiple sequence similarity display, these are rarely incorporated into genome databases. Visualization tools will benefit from a standard application programming interface to a large number of data sources, making their implementation more rewarding as they can find wider use.

### Simple access for humans and applications

The primary goal of any genome database is making data visible for human browsing. Nowadays, this is not enough. Applications need to access the data, as data mining needs to be automated. This is facilitated by standardized interfaces, which reduce the need to adapt tools to each individual data source. While local integration can rely on complex interfaces, public interfaces need to be simple so that they can be adopted easily and without detailed inside knowledge.

For local database and application integration, we are using standard design patterns like the business delegate pattern or model view controller to encapsulate application logic, data access and presentation. This drastically reduces the effort required for maintainability or for adding a new data source, analysis tool or view. Data is transported in XML format, which has the advantage of being easily transformed into other schemas, e.g. to comply to a standard public format for sharing.

Database interoperability between distributed centres via the Internet is hard to achieve through standardization. Defining standards that are accepted by all is tedious, especially in an academic

Copyright © 2003 John Wiley & Sons, Ltd. 

environment where experimentation is necessary. The BioMOBY project [7] was designed to reduce the need for standardization to a minimum, retaining flexibility, while achieving a maximum of integration. To achieve this, data schemas are lightweight, providing only basic functionality. However, objects can be overloaded to provide additional depth, which need not be utilized by every client. If this added functionality proves useful, it may evolve into a *de facto* standard. BioMOBY utilizes web services and a central registry that acts as a broker, removing the need for clients to know the data sources.

### Enabling comparative genomics

When data from multiple, distributed sources is easily accessible to a single client, the challenge becomes finding methods to compare data, e.g. between different species, and transferring knowledge, e.g. from a known gene to a predicted gene. While transferring function annotation to related protein sequences is common practice, new methods could enhance this by utilizing synteny information or tissue-specific expression. In-depth knowledge from the analysis of model species clearly needs to be exploited for less experimentally accessible organisms, but comparative analysis can also be a key to further interpretation of genome sequence.

### Conclusions

Data mining on heterogenous datasets requires easy accessibility of distributed data sources through standard interfaces. Data replication and warehousing can lead to problems in consistency and maintaining data up to date. We favour a federated approach, but this requires simple, standard interfaces to the distributed data sources, not only for human perusal but also for application access. While this requires some effort to create the required standards and infrastructure, in the long run this will allow more time to be dedicated to developing new data-mining tools instead of data collection and integration.

### References

1. Frishman D, Albermann K, Hani J, *et al*. 2001. Functional and structural genomics using PEDANT. *Bioinformatics* **17**: 44–57.
2. Haas BJ, Volfovsky N, Town CD, *et al*. 2002. Full-length messenger RNA sequences greatly improve genome annotation. *Genome Biol* **3**(6): RESEARCH0029.
3. Hubbard T, Barker D, Birney E, *et al*. 2002. The Ensembl genome database project. *Nucleic Acids Res* **30**: 38–41.
4. Sakata K, Nagamura Y, Numa H, *et al*. 2002. RiceGAAS: an automated annotation system and database for rice genome sequence. *Nucleic Acids Res* **30**: 98–102.
5. Schoof H, Zaccaria P, Gundlach H, *et al*. 2002. MIPS *Arabidopsis thaliana* database (MAtDB): an integrated biological knowledge resource based on the first complete plant genome. *Nucleic Acids Res* **30**(1): 91–93.
6. The *Arabidopsis* Genome Initiative. 2000. Analysis of the genome sequence of the flowering plant *Arabidopsis thaliana. Nature* **408**: 796–815.
7. Wilkinson MD, Links M. 2002. BioMOBY: An open source biological web services proposal. *Briefings in Bioinformatics* **3**(4): 331–341.

Copyright © 2003 John Wiley & Sons, Ltd. 
